# Impairment of Excitation-Contraction Coupling in Right Ventricular Hypertrophied Muscle with Fibrosis Induced by Pulmonary Artery Banding

**DOI:** 10.1371/journal.pone.0169564

**Published:** 2017-01-09

**Authors:** Yoichiro Kusakari, Takashi Urashima, Daisuke Shimura, Erika Amemiya, Genki Miyasaka, Shunsuke Yokota, Yoshitaka Fujimoto, Toru Akaike, Takahiro Inoue, Susumu Minamisawa

**Affiliations:** 1 Department of Cell Physiology, The Jikei University School of Medicine, Tokyo, Japan; 2 Department of Pediatrics, The Jikei University School of Medicine, Tokyo, Japan; 3 Department of Cardiac Surgery, The Jikei University School of Medicine, Tokyo, Japan; Texas A&M University Health Sciences Center, UNITED STATES

## Abstract

Interstitial myocardial fibrosis is one of the factors responsible for dysfunction of the heart. However, how interstitial fibrosis affects cardiac function and excitation-contraction coupling (E-C coupling) has not yet been clarified. We developed an animal model of right ventricular (RV) hypertrophy with fibrosis by pulmonary artery (PA) banding in rats. Two, four, and six weeks after the PA-banding operation, the tension and intracellular Ca^2+^ concentration of RV papillary muscles were simultaneously measured (n = 33). The PA-banding rats were clearly divided into two groups by the presence or absence of apparent interstitial fibrosis in the papillary muscles: F+ or F- group, respectively. The papillary muscle diameter and size of myocytes were almost identical between F+ and F-, although the RV free wall weight was heavier in F+ than in F-. F+ papillary muscles exhibited higher stiffness, lower active tension, and lower Ca^2+^ responsiveness compared with Sham and F- papillary muscles. In addition, we found that the time to peak Ca^2+^ had the highest correlation coefficient to percent of fibrosis among other parameters, such as RV weight and active tension of papillary muscles. The phosphorylation level of troponin I in F+ was significantly higher than that in Sham and F-, which supports the idea of lower Ca^2+^ responsiveness in F+. We also found that connexin 43 in F+ was sparse and disorganized in the intercalated disk area where interstitial fibrosis strongly developed. In the present study, the RV papillary muscles obtained from the PA-banding rats enabled us to directly investigate the relationship between fibrosis and cardiac dysfunction, the impairment of E-C coupling in particular. Our results suggest that interstitial fibrosis worsens cardiac function due to 1) the decrease in Ca^2+^ responsiveness and 2) the asynchronous activation of each cardiac myocyte in the fibrotic preparation due to sparse cell-to-cell communication.

## Introduction

Interstitial cardiac fibrosis frequently follows myocardial hypertrophy, which is commonly observed in patients and animal models with pressure-overloaded hearts due to conditions such as hypertension and stenotic valvular heart diseases [1–4]. In the right ventricle (RV), during the progression of pressure-overloaded cardiac hypertrophy, such as that seen in pulmonary arterial hypertension (PAH) patients, reactive interstitial fibrosis is initially observed as an adaptive response to increased wall stress without loss of cardiomyocytes [5–7]. Excess fibrosis eventually increases ventricular wall stiffness and causes diastolic dysfunction [7, 8]. It has been reported that cardiac fibrosis is an independent prognostic risk factor in patients with heart failure [9].

A growing body of evidence has demonstrated that impaired excitation-contraction (E-C) coupling plays an important role in cardiac dysfunction, especially diastolic dysfunction in the hypertrophic heart [10]. This suggests that impaired E-C coupling is a key mechanism responsible for cardiac muscle dysfunction. Myocardial fibrosis may, however, restrict the motion of myocytes and thereby impair overall cardiac function. Although the progression of interstitial fibrosis and the impairment of E-C coupling are hallmarks of diastolic dysfunction in hypertrophied cardiac muscle caused by pressure overload, the relationship between fibrosis and E-C coupling of the myocardium remains unclear under a hypertrophic condition. Direct measurements of myocardial stiffness, tension development, and intracellular calcium concentration in isolated muscle preparations are valuable to the understanding of integrated muscle function in hypertrophy and heart failure. In this study, an RV pressure-overloaded animal model enabled us to directly investigate the relationship between fibrosis and the impairment of E-C coupling in hypertrophied papillary muscles obtained from the RV. A preliminary report has already appeared in an abstract form [11].

## Method

### Solutions and chemicals

The composition (in millimolars) of Tyrode’s solution was NaCl, 136.9; KCl, 5.4; MgCl_2_, 0.5; NaH_2_PO_4_, 0.33; HEPES, 5; and glucose, 5; and pH was adjusted to 7.40 with NaOH [12].

### Pulmonary artery (PA)-banding procedure

All experiments were performed in accordance with the Guidelines on Animal Experimentation of The Jikei University School of Medicine (Tokyo, Japan). Experiments were performed after obtaining approval from the Animal Experiment Committee of the Jikei University School of Medicine (#26–051). The investigation conformed to the Guidelines for the Care and Use of Laboratory Animals published by the US National Institutes of Health. Sprague-Dawley male rats (n = 49, 6 weeks, 100–150 g) were purchased from Sankyo Laboratory (Tokyo, Japan). The PA-banding procedure was the same as previously reported [13]. Briefly, after intubation using Angiocath^TM^ 18G for rats, respiration was artificially managed under anesthesia with 2% isoflurane using a Harvard rodent ventilator (Harvard Apparatus, Holliston, MA), setting the respiratory rate at 120 to 140/min and tidal volume at 10 μl/g. After thoracotomy through the left 4th intercostal region, the thymus was removed and the main pulmonary artery was ligated twice with the outer tube of BD Angiocath^TM^20G (diameter, 1.88 mm) using 4.0 silk thread. The outer tube was then removed after double ligature and the thorax was closed. Age- and strain-matched controls (Sham, n = 24) were subjected to the same operative procedure with the sole exception of band placement. In preliminary experiments, we confirmed that the mean RV pressure in operated rats was significantly higher than that in Sham rats (66.7 ± 8.8 mmHg in operated rat RV pressure [n = 9] and 38.8 ± 5.2 mmHg [n = 8] in Sham, *p* < 0.05). In our preliminary analysis of the survival rate in 26 male rats with PA-banding using a log rank analysis of Kaplan-Meier curves, we found that 2 weeks after the PA-banding operation, the survival rate was relatively stable around 75%, and around 5 weeks after the PA-banding operation, the survival rate gradually decreased (S1 Fig). The survival rate of the control Sham-operated rats was 100% during the observation period. When we checked heart samples from rats that died around one or two weeks after the operation, the RV showed no hypertrophic change. In the present study, we selected 9 rats with PA-banding at 4 weeks and 16 rats with PA-banding at 6 weeks after the operation. After they were sacrificed, we did not find any pleural effusion in the chest cavity or ascites in the PA-banding group.

Animals were kept in cages with free access to food and drinking water. Animal(s) were euthanized by high dose of pentobarbital sodium (150 mg/kg, i.v.) when severe breathing difficulty or loss of activity was observed. Because the animals used in this study died without obvious or specific symptoms, they were not euthanized during the animal survival study. For the E-C coupling study, all rats that underwent the operation (PA-banding and Sham) were euthanized (total: 61 rats). For survival experiments, 9 rats that underwent the operation died without euthanasia and 17 rats that underwent the operation died with euthanasia. All rats were maintained at 22 ± 2°C under a 12-h lighting cycle following the National Institute of Health guidelines for animal experiments. We monitored the health of the animals every 12 h to check for unexpected severe breathing difficulty or loss of activity. We also touched animals gently to note unexpected changes in body temperature. We did not observe any unexpected severe breathing difficulty, loss of activity, or changes in body temperature before euthanization.

### Measurement of Ca^2+^ transient with aequorin using the aequorin method

Four to six weeks after the operation, rats were anesthetized with pentobarbital sodium (40–50 mg/kg, i.p.) and the hearts were quickly removed. After perfusion of the heart using the Langendorff apparatus, the RV wall was opened and thin papillary muscles were dissected out, as previously described for rabbit [14]. For measurement of the weights and biochemical factors, the ventricular walls were separated into three parts: the right and left free walls, and the septum.

After both ends of the papillary muscle preparation were tied with silk threads, the preparation was mounted horizontally between the fixed hook and the arm of a tension transducer. Electrical field stimulation was applied to the preparation using a pair of platinum electrodes placed in parallel to the preparation. To adjust muscle length (L) from slack to Lmax, at which active tension reached maximum, we stretched the papillary muscle at a rate of 0.05 to 0.1 mm/s and monitored the changes in resting and active tension. We measured the length and width of papillary muscles at Lmax. At Lmax, the sarcomere lengths of the preparation in the Sham group and the PA-banding group were almost identical, and this was confirmed by electron-microscopic analysis (S2 Fig). Cells on the upper surface of the preparation were penetrated and injected with a small volume of aequorin solution, and the aequorin light signals were detected and recorded simultaneously with tension. In the figures in the present study, the light signals were converted to [Ca^2+^]_i_ (intracellular Ca^2+^ concentration) as we previously described [14, 15]. Aequorin experiments were performed at 30.0 ± 0.5°C.

For an evaluation of twitch contraction, the following parameters were measured, as in previous studies [14, 15]: peak values of tension and Ca^2+^; time to peak tension and Ca^2+^ (the time measured from the onset of stimulus to the peak of tension and Ca^2+^, respectively); relaxation time (the time required for the tension to relax from 75% to 25% of the peak), and decay time of light (the time required for the light signal to decay from 75% to 25% of the peak).

### Membrane potential measurements

Conventional glass micro-electrodes filled with 3 mol/l KCl solution were used to measure the membrane potential, as described previously with slight modification [16] [17] [18]. Papillary muscles were mounted in a bath superfused with Tyrode’s solution (as described above) containing 20 mM/l BDM to suppress contraction. Papillary muscles were stimulated routinely at 0.1 Hz. Membrane potential in six or more sites in each preparation at Lmax was measured. The results presented were obtained from a single impalement.

### Histological assays of papillary muscles

After the experiments measuring tension and aequorin light signal, we fixed the preparations in 10% formalin. The midsection of the short-axis papillary muscle (4 μm) from the Sham group and the PA-banding group was obtained. To visualize fibrotic tissue, we stained the sections with Masson’s trichrome. The percent of fibrosis area (% of fibrosis) was calculated using ImageJ to quantify blue (fibrotic) vs. non-blue (non-fibrotic) pixels in the whole area of the mid-papillary and long-axis section. We found that % of fibrosis in Sham was less than 6.5% in this study. After calculation, all data obtained from PA-banding rats used in this study (general, E-C coupling, real-time polymerase chain reaction ([RT-PCR], and western blot) were divided into two groups by the presence (more than 6.5%) or absence (less than 6.5%) of apparent interstitial fibrosis in the papillary muscles: F+ or F- group, respectively. The cardiomyocyte cross-sectional area was also measured using ImageJ. At least 100 cardiomyocytes were examined in the short-axis section of each papillary muscle for a total of over 400 cardiomyocytes for each condition.

### Quantitative real-time polymerase chain reaction (RT-PCR)

The accumulation of PCR products was monitored in real time, and the crossing threshold (Ct) was determined with StepOne (Applied Biosystems, Foster City, CA). The relative change in gene expression was determined using the ΔΔCt method with normalization to GAPDH. Quantitative (Q) RT-PCR was performed with the primer sets listed in Table 1.

**Table 1:** List of primer sequences used for QRT-PCR.

Gene name	Forward primer	Reverse primer
TGF-β	ACCTGCAAGACCATCGACATG	CGAGCCTTAGTTTGGACAGGAT
CTGF	CAAGGACCGCACAGTGGTT	GCAGTTGGCTCGCATCATAG
Pro collagen I	CAGCGGAGAGTACTGGATCGA	CTGACCTGTCTCCATGTTGCA
Pro collagen III	TGCCATTGCTGGAGTTGGA	GAAGACATGATCTCCTCAGTGTTGA
TNF-α	AAAGCATGATCCGAGATGTG	AGCAGGAATGAGAAGAGGCT
ANP	TCGTCTTGGCCTTTTGGCT	TCCAGGTGGTCTAGCAGGTTCT
BNP	GGAAATGGCTCAGAGACAGC	AACAACCTCAGCCCGTCAC
GAPDH	TGGTGAAGCAGGCATCTGAG	TGCTGTTGAAGTCGCAGGAG
Cx43	CAAGGTGAAAATGAGGGG	AGACATAGGCGAGAGTGGAG

Transforming growth factor-β1 (TGF-β1); connective tissue growth factor (CTGF); procollagen I; procollagen III; tumor necrosis factor-alpha (TNF-α); atrial natriuretic peptide (ANP); brain natriuretic peptide (BNP); connexin 43 (Cx43); and glyceraldehyde 3-phosphate dehydrogenase (GAPDH) are shown.

### Immunohistochemistry

Formalin-fixed paraffin-embedded papillary tissue sections were subjected to a streptavidin–biotin–peroxidase complex assay (i-VIEW DAB kit; Ventana Japan, Yokohama, Japan) on the Ventana auto-immunostaining system (Ventana Japan). Slides were pretreated by the recommended procedures for antigen retrieval. The sections were then incubated with rabbit polyclonal antibody to connexin43/GJA1 (AbCam) at dilutions of 1:2000 for 30 min at room temperature and then washed three times in phosphate-buffered saline. Sections were incubated with the appropriate secondary antibody for 30 min at room temperature.

### Western blot analysis

Hearts were harvested, snap frozen, and crushed in liquid nitrogen. The tissue was then homogenized in cold lysis buffer, as in previous research [19]. Protein concentration was measured by the Bradford method (Bio-Rad, Hercules, CA). Sodium dodecyl sulfate-polyacrylamide gel electrophoresis (SDS-PAGE) was performed under reducing conditions on 4 to 20% gradient gels. Proteins were transferred to a nitrocellulose membrane. Blots were incubated with primary antibodies for 18 to 20h at 4°C. Blots were then incubated with horseradish peroxidase-conjugated secondary antibody, and the signal was detected using enhanced chemiluminescence (Cell Signaling Technology). The primary antibodies used in the present study were: GAPDH (2118, rabbit monoclonal antibody, Cell Signaling Technology), connexin 43 (11370, rabbit polyclonal antibody, AbCam), anti-troponin I (4002, rabbit polyclonal antibody, Cell Signaling Technology), anti-phosphorylated troponin I (serine 23/24) (4004, rabbit polyclonal antibody, Cell Signaling Technology), anti-SERCA2a (1314, mouse monoclonal antibody, Sigma, Japan), anti PLN (A010-14, mouse monoclonal antibody, Badrilla, United Kingdom), anti-phosphorylated PLN (serine 16 or threonine 17) (A010-12, rabbit polyclonal antibody, Badrilla, United Kingdom), anti-RyR2 (kindly provided by Dr. Andrew Marks of Columbia University, USA), anti-phosphorylated RyR2 (kindly provided by Dr. Andrew Marks of Columbia University, USA), and anti-NCX1 (kindly provided by Dr. Takahiro Iwamoto of Fukuoka University, Japan).

### Statistical analysis

**Fig 1:**
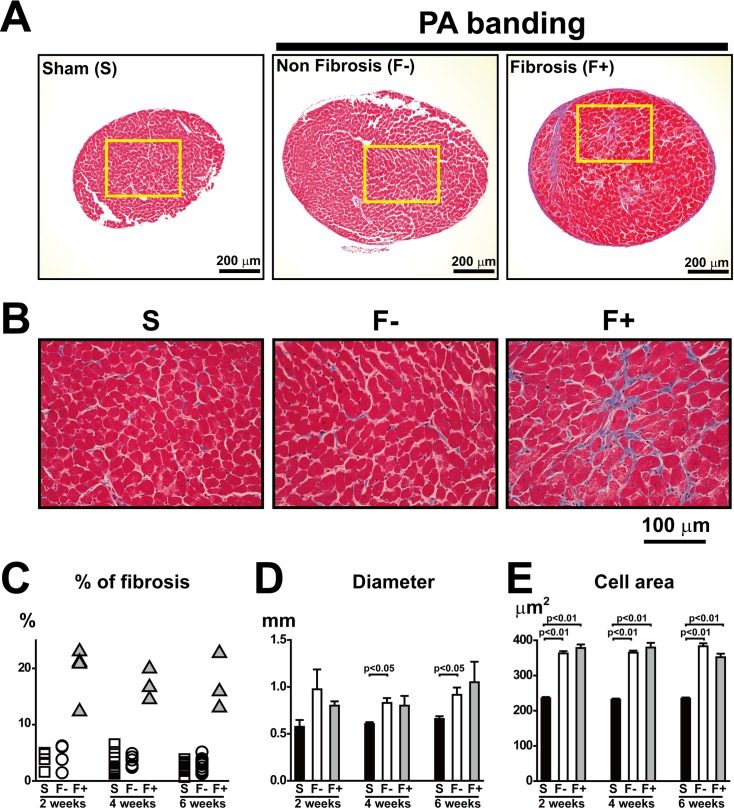
Morphology and histology in papillary muscle from rats with Sham and PA banding. A and B: Representative photos of Masson’s trichrome staining in mid-papillary short-axis sections from Sham and PA-banding 4 weeks after the operation. The yellow rectangle in A indicates the area of enlarged images shown in B. Rats with PA-banding were divided into F- (hypertrophy without interstitial fibrosis) and F+ (hypertrophy with interstitial fibrosis). C and D: Quantitative analysis of the interstitial fibrosis area examined by Masson’s trichrome staining (C), and muscle diameter in papillary muscles (D). Values are means ± SE; n = 4 in Sham, n = 4 in F-, and n = 4 in F+ 2 weeks after PA-banding; n = 12 in Sham, n = 6 in F-, and n = 3 in F+ 4 weeks after PA-banding; n = 12 in Sham, n = 13 in F-, and n = 3 in F+ 6 weeks after PA-banding. E: Analysis of cell area in papillary muscles 2, 4, and 6 weeks after the operation. The surface areas of over 400 cells from 3 to 5 papillary muscles of individual rats in each group were measured.

**Fig 2:**
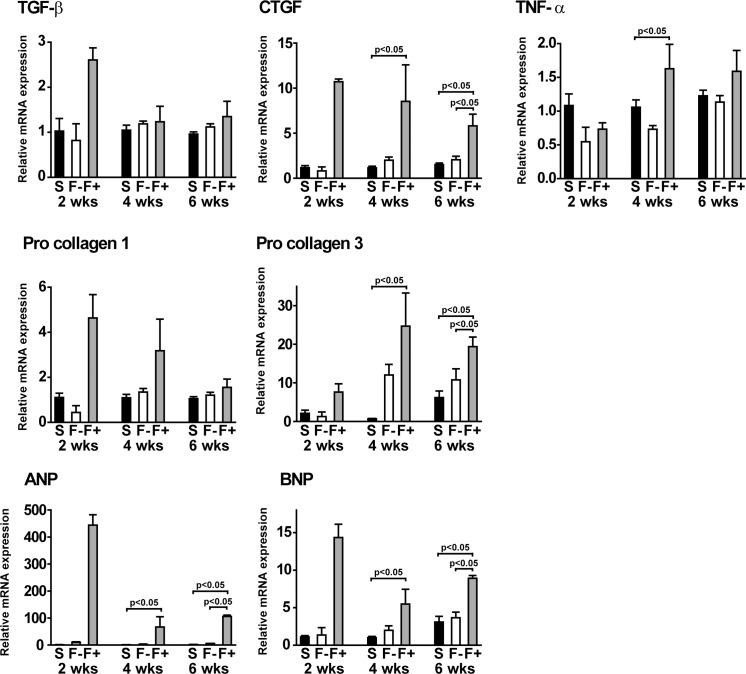
Expression levels of pro-fibrotic factors and heart stress markers in the hypertrophy with fibrosis group. Expression levels of mRNA from the right ventricle were measured by QRT-PCR. Summarized data of transforming growth factor beta (TGF-β), connective tissue growth factor (CTGF), pro-collagen I, pro-collagen III, tumor necrosis factor-alpha (TNF-α), atrial natriuretic peptide (ANP), and brain natriuretic peptide (BNP). Values are means ± SE; n = 4 in Sham, n = 4 in F-, and n = 4 in F+ 2 weeks after PA-banding; n = 12 in Sham, n = 6 in F-, and n = 3 in F+ 4 weeks after PA-banding; n = 12 in Sham, n = 13 in F-, and n = 3 in F+ 6 weeks after PA-banding.

Data are presented as means ± standard error of the mean (SEM). Papillary muscle diameter, RT-PCR data, and general characteristics in Table 2 and Figs 1 and 2 were tested for significance with the nonparametric test with multiple comparisons (Steel-Dwass method). For other multiple comparisons, one-way analysis of variance (ANOVA) with the Bonferroni post hoc test was used. For all analyses, a *p* value of <0.05 was considered significant.

**Table 2:** General parameters in tissues from sham (Sham), hypertrophy without fibrosis (F-), and hypertrophy with fibrosis (F+).

**Time after operation (2 weeks)**			
**2 weeks**	**Sham (n = 4)**	**F- (n = 4)**	**F+ (n = 4)**
**Body weight (g)**	283.75 ± 8.03	364.13 ± 46.38	278.00 ± 8.83
**Heart weight / TL (g/cm)**	269.50 ± 9.17	339.77 ± 52.39	356.31 ± 7.10
**RV weight / TL (mg/cm)**	32.75 ± 0.68	58.66 ± 11.70	73.84 ± 2.44
**RV/(LV+SepW)**	0.19 ± 0.01	0.29 ± 0.03	0.43 ± 0.03
**Lung weight / TL (mg/cm)**	297.40 ± 6.41	383.43 ± 17.41	436.12 ± 64.08
**Liver weight / TL (g/cm)**	3.03 ± 0.09	3.67 ± 0.22	4.20 ± 0.98
**TL (cm)**	4.36 ± 0.08	4.43 ± 0.09	4.33 ± 0.18
**Time after operation (4 weeks)**			
**4 weeks**	**Sham (n = 12)**	**F- (n = 6)**	**F+ (n = 3)**
**Body weight (g)**	323.29 ± 13.64	407.75 ± 13.59*	478.00 ± 21.59*
**Heart weight / TL (g/cm)**	312.98 ± 10.47	312.06 ± 16.57	442.67 ± 44.24*
**RV weight / TL (mg/cm)**	41.32 ± 1.50	60.57 ± 4.30**	81.94 ± 6.65*
**RV/(LV+SepW)**	0.21 ± 0.01	0.32 ± 0.04*	0.41 ± 0.03*
**Lung weight / TL (mg/cm)**	297.40 ± 6.41	383.43 ± 17.41**	436.12 ± 64.08*
**Liver weight / TL (g/cm)**	3.03 ± 0.09	3.67 ± 0.22*	4.20 ± 0.98*
**TL (cm)**	4.36 ± 0.08	4.43 ± 0.09	4.33 ± 0.18
**Time after operation (6 weeks)**			
**6 weeks**	**Sham (n = 12)**	**F- (n = 13)**	**F+ (n = 3)**
**Body weight (g)**	425.88 ± 12.36	452.27 ± 6.88	342.67 ± 31.99^♥^
**Heart weight / TL (g/cm)**	300.55 ± 8.01	327.04 ± 9.90	387.27 ± 49.22
**RV weight / TL (mg/cm)**	40.54 ± 0.97	53.40 ± 4.35^♦^	96.87 ± 11.15^♦^^♥^
**RV/(LV+SepW)**	0.21 ± 0.01	0.27 ± 0.02	0.54 ± 0.02^♦^^♥^
**Lung weight / TL (mg/cm)**	323.17 ± 13.41	330.14 ± 8.73	283.84 ± 34.23
**Liver weight / TL (g/cm)**	3.67 ± 0.21	3.91 ± 0.14	3.14 ± 0.52
**TL (cm)**	4.66 ± 0.04	4.76 ± 0.04	4.60 ± 0.06

*: *p* < 0.05 vs. Sham (4 weeks)

**: *p* < 0.01 vs. Sham (4 weeks).

^♦^: *p* < 0.05 vs. Sham (6 weeks)

^♥^: *p* < 0.01 vs. F- (6 weeks).

## Results

### Development of fibrosis in the PA-banding group

We found that the PA-banding procedure produced RV hypertrophy in all animals examined. The ratio of RV weight to septum and LV weight in both F- and F+ were significantly higher than that in Sham at 4 and 6 weeks after the operation (Table 2). Interestingly, the interstitial fibrotic area was obvious in some samples of PA-banding heart, as shown in Fig 1A and 1B and S3 Fig. To investigate the E-C coupling parameters of the hypertrophied myocardium, we divided rats with PA banding into two groups as described in Method: one group in which the fibrotic area was greater than 13% (interstitial fibrotic group; F+) and another group in which the fibrotic area was less than 6.5% (non-fibrotic group; F-), as shown in Table 3. The % of fibrosis in F- was almost the same as that in Sham (Fig 1C and S4 Fig).

**Table 3:** Data set of tension and Ca^2+^ in papillary muscles using the aequorin method.

**Sham (n = 28)**	**Papillary muscle**			**Peak value**		**Peak time**	
	**% of fibrosis**	**Length (mm)**	**Width (mm)**	**Tension (mN)**	**Ca**^**2+**^ **(μM)**	**Tension (ms)**	**Ca**^**2+**^ **(ms)**
**2 weeks**	1.5	3.5	0.6	12.8	1.9	126	23
	1.6	3.4	0.6	8.3	1.8	106	20
	4.8	3.3	0.8	15.9	1.8	106	20
	3.9	2.3	0.4	8.5	1.4	103	23
**4 weeks**	6.5	4.8	0.6	6.7	1.6	105	25
	2.6	3.6	0.7	14.8	1.6	124	24
	2.1	2.7	0.6	14.6	1.9	125	21
	4.1	2.5	0.6	9.9	1.2	132	27
	1.4	2.2	0.6	4.5	1.2	116	28
	2.2	2.7	0.5	10.6	1.8	124	24
	5.1	3.2	0.8	17.2	1.5	134	27
	1.5	2.6	0.7	6.5	1.2	117	31
	1.8	2.4	0.6	8.3	1.3	111	23
	1.3	2.2	0.5	7.0	1.6	119	23
	3.3	3.0	0.7	9.4	1.7	115	23
	1.5	2.8	0.7	12.1	1.7	126	23
**6 weeks**	0.7	2.5	0.5	10.2	1.4	132	21
	1.8	3.4	0.7	15.4	1.7	127	26
	3.9	2.5	0.6	8.9	1.4	122	23
	2.9	2.5	0.7	11.4	1.1	126	22
	2.3	2.5	0.9	13.3	1.7	120	31
	2.1	2.5	0.7	13.0	2.1	126	27
	2.5	3.5	0.7	19.5	1.6	125	20
	3.2	2.8	0.6	14.0	2.4	122	30
	3.4	3.7	0.8	18.3	2.3	128	29
	2.2	3.8	0.7	20.9	1.5	127	26
	2.3	3.3	0.6	17.0	1.9	133	25
	3.1	2.8	0.6	18.9	1.7	146	29
**PAB (n = 33)**	**Papillary muscle**			**Peak value**		**Peak time**	
	**% of fibrosis**	**Length (mm)**	**Width (mm)**	**Tension (mN)**	**Ca**^**2+**^ **(μM)**	**Tension (ms)**	**Ca**^**2+**^ **(ms)**
**2 weeks**	5.0	4.7	1.6	13.6	4.0	132	27
	1.6	2.9	0.8	13.3	2.3	110	24
	4.8	2.6	0.7	12.5	1.9	121	25
	3.9	4.0	0.9	14.7	2.6	119	26
	22.5	2.9	0.7	7.4	2.8	166	48
	22.3	3.6	0.9	6.5	4.5	157	45
	20.8	3.8	0.8	5.3	2.1	172	55
	28.4	3.2	0.9	9.3	2.5	162	43
**4 weeks**	2.4	3.2	0.7	16.6	2.3	125	27
	2.6	3.2	1.0	27.5	2.2	138	27
	3.7	3.6	0.9	26.6	2.4	144	26
	2.8	2.5	1.0	12.8	1.7	118	27
	4.3	2.1	0.8	15.3	1.6	133	28
	4.9	3.4	0.8	12.4	1.5	122	27
	20.0	3.0	0.8	7.1	2.3	177	50
	14.5	2.9	1.0	5.7	2.4	148	48
	16.7	2.5	0.7	12.3	2.3	178	52
**6 weeks**	3.2	3.1	1.7	23.7	2.8	138	29
	2.0	4.2	1.0	20.1	3.5	126	31
	2.9	2.5	0.8	11.8	2.1	120	26
	3.8	3.5	1.0	18.2	1.9	122	34
	3.4	2.7	0.7	11.1	1.4	118	25
	1.9	3.0	1.1	9.0	2.0	187	28
	1.5	3.8	0.7	21.1	1.8	104	25
	1.3	4.0	1.1	19.5	1.9	178	28
	5.1	3.0	0.7	14.3	1.8	129	28
	2.5	3.0	0.7	18.7	2.1	131	29
	1.8	3.2	0.8	14.8	1.6	134	30
	3.7	3.2	0.8	15.5	1.2	131	31
	3.9	3.2	0.8	13.7	1.9	126	29
	15.9	3.8	1.5	12.4	1.4	187	43
	13.0	3.5	1.0	7.3	3.5	178	47
	22.7	3.8	0.7	8.8	2.5	179	48

The percent of fibrosis, length, width, peak value of tension, peak value of Ca^2+^, time of peak tension, and time of peak Ca^2+^ in papillary muscles from Sham-operated rats (n = 28) and PA-banding rats (n = 33). Note that the % of fibrosis was clearly different.

Importantly, there was no significant difference in the width of the papillary muscle between F- and F+, which was significantly larger than that in Sham (Fig 1D). The length of the papillary muscle did not vary among Sham, F-, and F+ groups. Furthermore, we found that the cell area in the mid-papillary section of the short axis was larger in F- and F+ than in Sham and that there was no difference in cell area between F- and F+ (Fig 1E). Taken together, both F- and F+ groups exhibited almost identical hypertrophic changes in the RV.

### Pro-fibrotic factors and heart failure markers were increased in F+

To confirm whether the fibrotic change appeared at the transcriptional level, we investigated the mRNA expression levels of pro-fibrotic factors. Although the expression level of transforming growth factor beta (TGF-β) in F+ did not increase significantly, the expression levels of connective tissue growth factor (CTGF) and pro-collagen III mRNAs in F+ were significantly higher than those in Sham at 4 and 6 weeks (Fig 2). Moreover, the expression level of TNF-α mRNA at 4 weeks was significantly higher in F+ than that in Sham. In addition, we found that the expression levels of ANF and BNP mRNAs, which are well established as stress markers in the heart, were significantly higher in F+ than in Sham and F- at 4 and 6 weeks. Although we did not find a statistically significant change, the expression levels of ANF and BNP mRNA were extremely high in F+ at 2 weeks. These morphological, histological, and gene expression data revealed that the papillary muscle samples in the pressure-overloaded hearts consisted of two distinct groups, a fibrosis group and a non-fibrosis group, with a similar extent of muscle hypertrophy. Further analysis was thus performed to compare the effect of fibrosis on tension development and intracellular calcium transient among Sham, F-, and F+ groups.

### Passive stiffness was increased in F+

**Fig 3:**
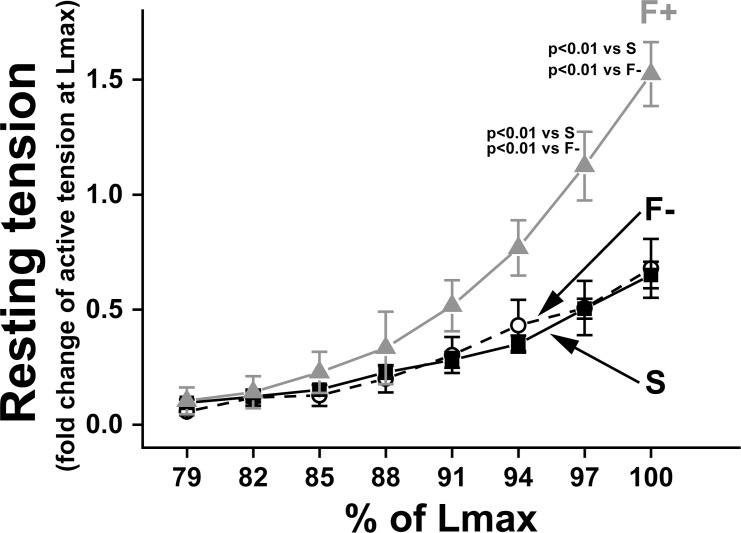
Myocardial stiffness in papillary muscle with and without fibrosis. The relationship between muscle stretch and resting tension in papillary muscle. On the x-axis, each muscle length was normalized by Lmax, at which papillary muscle developed maximum active tension. On the y-axis, muscle resting tension was normalized by maximum active tension at Lmax. Filled squares: Sham; open circles: F- (hypertrophy); and gray triangles: F+ (hypertrophy with fibrosis). Values are means ± SE; n = 21 in Sham, n = 17 in F-, and n = 4 in F+.

To evaluate myocardial stiffness in papillary muscle, we measured the increase in resting tension when we stretched the preparation from slack length to Lmax, at which active tension was maximum. Resting tension in F+ was significantly higher than in Sham and F- at 97% and 100% Lmax (Fig 3), indicating that passive stiffness was increased in F+ (hypertrophied muscle with fibrosis). In this study, F- (hypertrophied muscle without fibrosis) did not show any changes in stiffness compared with that in Sham, as in a previous report [1]. These data indicate that there is a close relationship between fibrotic change and muscle stiffness as we could expect.

### E-C coupling was impaired in hypertrophic papillary muscles with fibrosis

**Fig 4:**
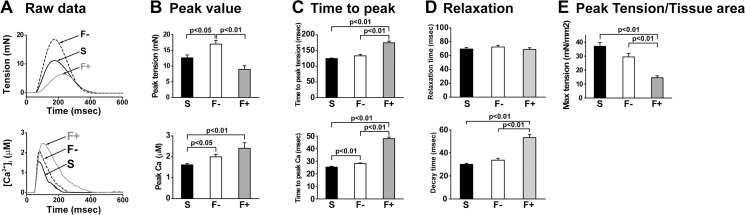
Excitation-contraction coupling in papillary muscle with and without fibrosis. Tension and Ca^2+^ transients in papillary muscles simultaneously measured by the aequorin method. A: Representative traces in tension (upper) and intracellular Ca^2+^ (lower) in each sample. B: Summarized data of peak value in each sample. C: Summarized data of the peak time of tension and intracellular Ca^2+^. D: Summarized data of relaxation time. E: Summarized data of tension divided by the cross-sectional area of the muscle in each sample.Values are means ± SE; n = 28 in Sham (S), n = 23 in hypertrophy without fibrosis (F-), and n = 10 in hypertrophy with fibrosis (F+).

Next, we analyzed and compared the parameters related to E-C coupling. The most significant change in E-C coupling in accordance with cardiac fibrosis was a decrease in maximal tension development. The peak value of tension was significantly lower in F+ than in F- (Fig 4A and 4B upper). There was no significant difference in the peak value of tension between Sham and F+, but the tension in F+ was slightly lower than in Sham. In contrast, the peak value of intracellular Ca^2+^ concentration was higher in F- and F+ than in Sham (Fig 4A and 4B lower). The time to peak tension was significantly prolonged in F+ compared to Sham and F- (Fig 4C upper). In addition, F+ showed a significantly longer time to peak Ca^2+^ when compared with Sham and F- (Fig 4C lower). In the relaxation phase, the relaxation time of tension did not differ among Sham, F-, and F+; however, the decay time of Ca^2+^ signal in F+, which showed Ca^2+^ removal time from the cytosol, was significantly longer than in Sham and F- (Fig 4D). We found that the time to peak Ca^2+^ had the highest correlation coefficient to % of fibrosis among other parameters (Table 4). Tension divided by cross-sectional area of the muscle was shown in Fig 4E, and F+ was significantly lower than Sham and F-.

**Table 4:** Correlation coefficient of physical and biological data to % of fibrosis.

Parameter	*r* value
**Time to peak Ca**^**2+**^	0.93
**Decay time**	0.70
**RV/(LV+SepW)**	0.70
**RV/LV**	0.69
**Time to peak tension**	0.66
**HW/BW**	0.64
**RV weight (mg)**	0.62
**HW/TL**	0.59
**Peak tension**	-0.59

Highly correlated parameters in body, tissues, tension, and Ca^2+^ are shown. Time to peak Ca^2+^ was very highly correlated to % of fibrosis.

**Fig 5:**
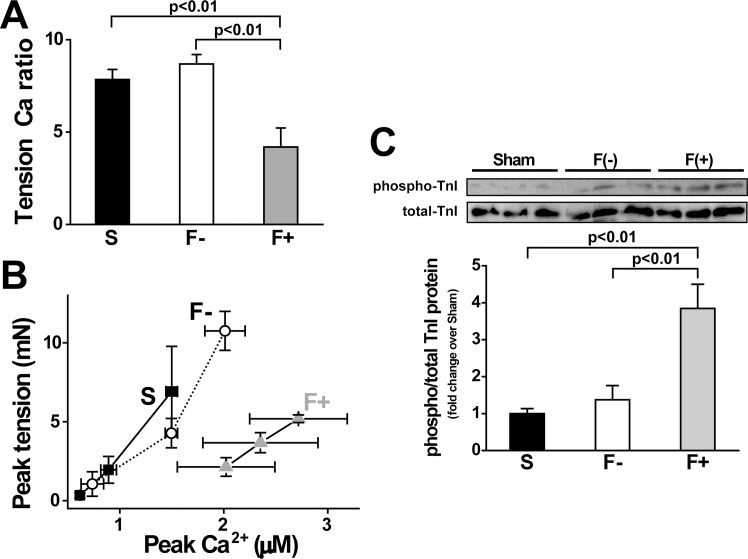
Relationship between tension and Ca, and phosphorylation level of TnI. A: Tension Ca^2+^ ratio. Peak values of tension divided by peak values of Ca^2+^ in each sample from Table 3. Values are means ± SE; n = 24 in Sham (S), n = 19 in hypertrophy without fibrosis (F-), and n = 6 in hypertrophy with fibrosis (F+). B: Relationship between peak tension and peak Ca^2+^ under various extracellular Ca^2+^ solutions. We plotted a series of changing peak tensions and peak Ca at different extracellular Ca^2+^ concentrations (0.5, 1, and 2 mM). Values are means ± SE; n = 5 each. Peak plot in F+ shows a right-ward shift compared with S and F-. C and D: Western blots of phosphorylation of TnI (Ser23/24) and total TnI. Representative blot photo (C) and summarized data (D). Values are means ± SE; n = 6 each.

Measurements of tension and Ca^2+^ revealed that the relationship between tension and Ca^2+^ in F+ differed from that in Sham and F-. Therefore, we compared the ratio of peak tension to peak Ca^2+^, and found that the ratio in F+ was significantly reduced (Fig 5A). To evaluate the Ca^2+^ responsiveness of tension, we plotted peak intracellular Ca^2+^ and peak tension when extracellular Ca^2+^ concentration was changed in a stepwise manner (Fig 5B), as in a previous study [20]. The relationship between tension and Ca^2+^ in F+ obviously shifted towards to the right in comparison with that in Sham and F-, indicating that the Ca^2+^ responsiveness of contractile protein in F+ was lower than that in Sham and F-. Since the phosphorylation of troponin I (TnI) is known to decrease the Ca^2+^ responsiveness of the contractile elements [21], we measured the phosphorylation level of TnI and found that it was significantly greater in F+ than that in Sham and F- (Fig 5C).

### Protein expression levels of Ca^2+^ handling factors

**Fig 6:**
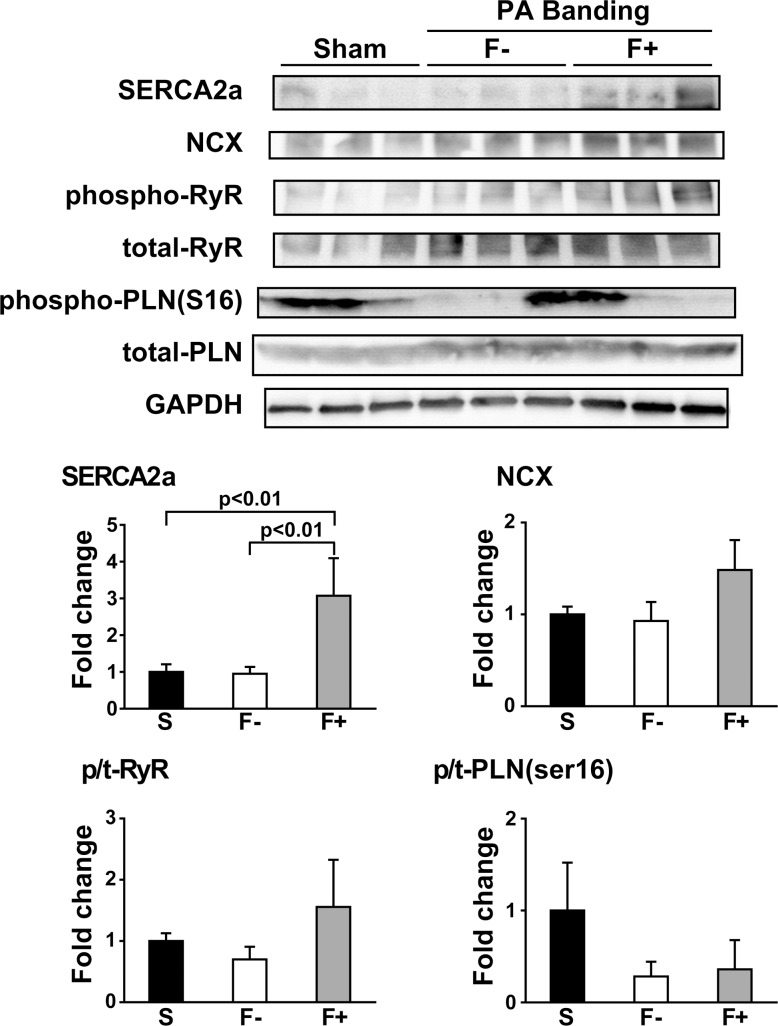
Expression of Ca^2+^ handling proteins in hypertrophy with fibrosis. Expression levels of SERCA2a, NCX, phospho-RyR, total-RyR, phospho-PLN (ser16), and total-PLN protein measured by the western blot technique. Values are means ± SE; n = 6 each.

For further estimation of the effect of fibrosis in E-C coupling, we measured the expression levels of Ca^2+^ handling proteins using the western blot technique. The expression level of SERCA2a in F+ was significantly higher than that in Sham and F-. However, the expression level of NCX and the phosphorylation levels of RyR and phospholamban (PLN) were not significantly changed among Sham, F-, and F+ (Fig 6).

### Prolonged action potential duration in fibrosis

**Fig 7:**
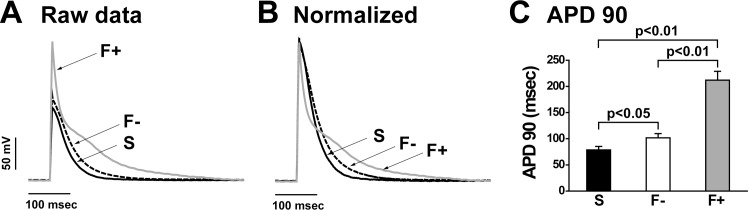
Action potential duration was prolonged in hypertrophy with fibrosis. A and B: Representative membrane potential traces (A; raw and B; normalized) in each sample. Six or more sites in each papillary muscle at Lmax were measured. The results presented originated from a single impalement. C: Summarized data of action potential duration (APD) from 3 individual papillary muscles in each group. APD90 (the APD at 90% of repolarization) was analyzed. Values are means ± SE; n = 3 each.

Next, we investigated the action potential duration (APD) of the papillary muscle in each preparation to confirm how action potential was influenced by fibrosis, because fibrosis of cardiac tissue reportedly influences the APD, refractoriness, and conduction velocity in animal and mathematical models [22, 23]. Typical traces of raw data and normalized data of membrane potential in each sample indicates that the APD in F+ was extremely prolonged. In summarized data, the APD90 (APD at 90% of repolarization) in F+ was significantly longer than that in Sham and F- (Fig 7).

### Reduced connexin 43 expression in fibrosis

**Fig 8:**
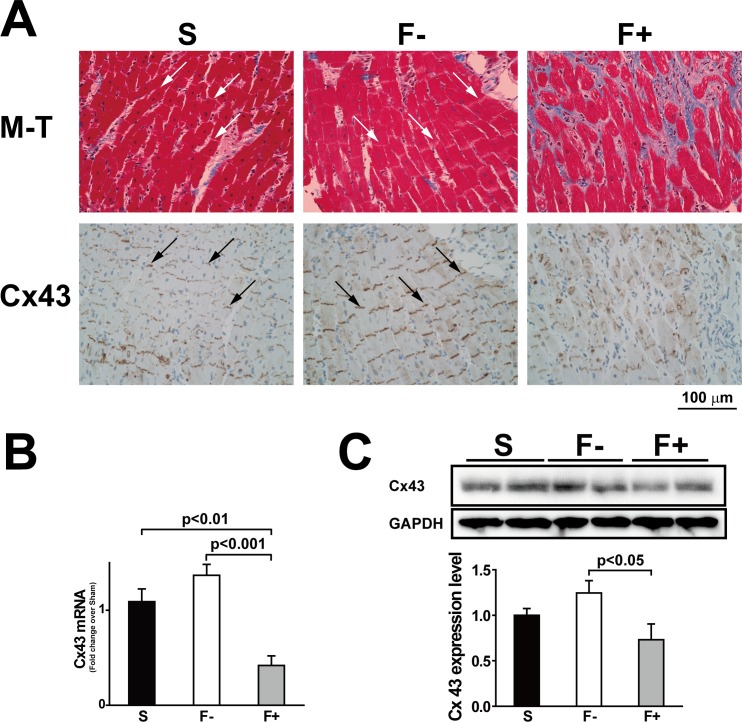
Reduced intercalated disks and connexin 43 expression in hypertrophy with fibrosis. A: Representative photos of Masson’s trichrome staining (M-T; upper) and connexin 43 immunohistochemistry staining (Cx43; lower) in the long-axis sections of papillary muscles obtained from Sham (S), hypertrophy without fibrosis (F-), and hypertrophy with fibrosis (F+). Arrows represent intercalated disks. In F+, intercalated disks were invaded with fibrosis and the Cx43 expression level was reduced. B; Expression of Cx43 mRNA was measured by QRT-PCR. Values are means ± SE; n = 21 in Sham, n = 19 in F-, and n = 6 in F+. C: Expression of Cx43 proteins was measured by the western blot technique. Values are means ± SE; n = 6 each.

When histological changes were observed in a long-axis section, the intercalated disks in Sham and F- were clearly organized, however, those in F+ were disorganized (Fig 8A). We further investigated the expression pattern of connexin 43 (Cx43) protein using a Cx43 antibody since Cx43 was one of the main proteins constructing the gap junction in ventricular tissues [24, 25]. The expression of Cx43 protein in F+ almost disappeared, especially in the interstitial fibrosis area. In contrast, F- showed slightly stronger expression levels of Cx43 compared with Sham, which was supported by the expression levels of mRNA and protein (Fig 8B and 8C).

## Discussion

In the present study, we demonstrated that the impaired E-C coupling was an underlying mechanism of the depressed tension development in the papillary muscle with interstitial fibrosis in PA-banding rats. To date, many researchers have reported that interstitial cardiac fibrosis follows pathological conditions such as PAH, ischemia, hypertrophy, and cardiomyopathy and that it is related to cardiac dysfunction [7, 19, 26, 27]. However, few reports have directly investigated how cardiac fibrosis affects E-C coupling because there are no adequate animal models. In this study, we could clearly show the differences in E-C coupling in the presence or absence of fibrosis. The PA-banding model has two significant advantages. First, our model provides papillary muscles that could be clearly divided into a fibrotic or non-fibrotic group, and the biochemical parameters obtained from the RV free wall support this classification. Second, the aequorin method enables us to simultaneously measure the tension and intracellular Ca^2+^ concentration of multicellular preparations (papillary muscle) that contains fibrotic transition tissues. One of the advantages of using papillary muscle is that we can evaluate muscle contraction and Ca^2+^ signals from the cells that maintain cell-to-cell communication in the tissue, which is difficult in isolated single cell preparations.

In this study, we used papillary muscle as an indicator of fibrotic change and measurement of E-C coupling. After measuring the E-C coupling, we fixed the papillary muscles for the Masson trichrome stain, and then divided PA-banding rats in terms of the percent of fibrosis size in papillary muscles. Since we used all of the subject animals, there was no selection bias.

### Decrease of contraction in papillary muscle with fibrotic change induced by pressure overload

Fibrotic and hypertrophic papillary muscle showed significantly higher stiffness (Fig 3). However, non-fibrotic and hypertrophic papillary muscle, which had an identical extent of hypertrophic change as found in F+, showed similar stiffness to that of non-fibrotic and non-hypertrophic papillary muscle (Sham). This finding indicates that hypertrophy itself does not affect muscle stiffness, which supports the result of Conrad et al. [1].

The most striking finding in the present study is that the peak tension was decreased in fibrotic papillary muscles, even though the peak Ca^2+^ was increased. The relationship between Ca^2+^ and tension in F- was similar to that in Sham, however, the relationship in F+ was clearly shifted to the right. These results indicate that the Ca^2+^ responsiveness in F+ was much lower than that in Sham and F-. This suggests that cardiac dysfunction in the heart with fibrosis is caused by the decreased Ca^2+^ responsiveness, not by the impairment of Ca^2+^ supply from the sarcoplasmic reticulum. One of the intracellular mechanisms of the lower Ca^2+^ responsiveness of F+ is the higher phosphorylation level of TnI compared to that of Sham and F- preparations. Phosphorylation of TnI is known to decrease Ca^2+^ affinity of troponin C (TnC) which leads to lower contraction [21]. If Ca^2+^ binding to TnC is decreased, Ca^2+^ is easily released from the Ca^2+^ binding site of TnC, which could explain the larger peak of Ca^2+^ transient and lower contraction in F+. The slower decay time of the Ca^2+^ transient in F+ might be due to slower Ca^2+^ removal from the cytosol. Since the phosphorylation level of PLN and RyR2 was not significantly altered among Sham, F-, and F+, phosphorylation of these Ca^2+^-binding mechanisms cannot explain the slower time course of the Ca^2+^ transient. We also calculated the ratio of PLN/SERCA in these samples as an activity of SERCA. We found that F- was 2.2-fold and that F+ was 1.7-fold of the PLN/SERCA ratio compared with Sham. The upregulation of SERCA2a in F+ might be a compensative response to higher intracellular Ca^2+^.

Although troponin I is known to be phosphorylated by protein kinase A (PKA), which also phosphorylates RyR2 and PLN in the heart, we found that the phosphorylation levels of RyR2 and PLN were not increased. This indicates that fibrosis selectively increases the phosphorylation level of troponin I. Szymanska et al. reported that the responses to adrenergic stimulation differed between PLN and troponin I [28]. Kirchhefer et al. reported that protein phosphatase 2A activity differed among RyR, PLN, and troponin I [29]. Kuo et al. showed that reduced expression of polycystin 2, which senses fluid stress, increased the phosphorylation level of cardiac troponin I with a reduced phosphorylation level of PLN [30]. Moreover, ablation of the troponin I phosphorylation site reduced papillary muscle stiffness [31]. It is intriguing to consider that the increased mechanical stiffness due to fibrosis may promote selective stress on myofilaments, which induces troponin I phosphorylation. Further investigation is required to prove this hypothesis.

The APD90 was extremely prolonged in fibrotic papillary muscles. Longer APD is expected to enhance Ca^2+^ influx [32]. To elucidate the mechanisms of the higher and longer action potential in fibrotic papillary muscles, we measured the expression level of Na_v_1.5 mRNA, and found that it was slightly reduced but not significantly changed in comparison with Sham (S5 Fig). Although the molecular mechanisms of the altered channel activities are not yet clarified, the prolongation of APD, in particular at the tail of the action potential in F+, might partly contribute to the enhanced and prolonged Ca^2+^ transient.

### Cell-to-cell communication in F+

Cx43 is a component in gap junctions that connects myocytes and is thought to have a pivotal role in the synchronized contraction of the heart [24, 33, 34]. Our immunohistochemical study revealed that Cx43 protein almost disappeared, especially in the fibrotic area (Fig 8A). Thus cell-to-cell communication is disturbed by fibrosis that appears around intercalated disks. Loss of synchronous activation of cardiac cells, even if the myocytes function is preserved in F+, might be related to low contractility and longer time to peak Ca^2+^, despite the higher peak Ca^2+^ in fibrotic papillary muscles.

### Limitations of this study

It is important to measure the sarcomere length of papillary muscles at Lmax, however, the measurement is technically very difficult. Laser diffraction could not be adapted to measurements of sarcomere length in papillary muscles, because of muscle thickness (over 500 μm). Instead, we tried to measure sarcomere length using electron microscopy (EM). After measurement of the tension with Ca^2+^, we fixed the papillary muscles with 10% glutaraldehyde, keeping the muscle length at Lmax for 10 min. Then, we measured these papillary samples from PAB groups by EM and found that the sarcomere length of both samples was almost 2.2 μm (n = 2). We could not, however, identify whether these PAB samples were obtained from F- or F+, because they were not stained with Masson trichrome. We assumed that one sample belonged to the F+ group and that the other belonged to the F- group, because the former showed a much longer peak Ca time than the latter. Further investigation is required to demonstrate that the sarcomere length of F- and F+ is almost equal.

The data of papillary muscles might not closely correlate with the data of the RV free wall. As shown in Fig 1D, papillary muscle diameter was almost identical between F- and F+, although RV weight was heavier in F+ than that in F- (Table 2). Fibrotic change might not match between papillary muscle and the RV free wall. As shown in Fig 2, the significant increases in ANP and BNP levels in F+ suggest that the rats in F+ were exposed to severe afterload when compared with those in F-. These data suggest that papillary muscles might be more sensitive to hypertrophic stimuli than the RV free wall. Further investigation is required to confirm this assumption. Despite these facts, we believe that our results clearly showed that fibrosis played a critical role in the impairment of E-C coupling as a result of pressure-overloaded stimuli.

## Conclusions

The PA-banding model employed in this study is useful for the investigation of the development of fibrosis and its influences on papillary muscle contractile properties, because the papillary muscle of this model can quickly change from the adaptive state to the maladaptive state of heart functions during the course of PA banding. Our results support the view that fibrosis, not hypertrophy, is a critical factor in cardiac dysfunction due to pressure overload.

Dysfunction of hypertrophied myocardium with fibrosis can be explained by the following mechanisms: 1) the lower Ca^2+^ responsiveness of the contractile elements that is attributable to TnI phosphorylation and 2) the disturbance of synchronous activation in each myocyte induced by impaired cell-to-cell communication.

Further investigation is required to identify the factors that promote fibrosis and alter the E-C coupling mechanisms.

## Supporting Information

S1 Fig Survival rate of PA-banding. The Kaplan-Meier curve of PA-banding and Sham-operated rats is shown. The survival rate of rats with PA-banding was significantly lower than that of Sham-operated rats.
(TIF)

S2 Fig Electron microscope images in Sham and PA-banding. Representative electron microscope images are shown. At Lmax, the sarcomere lengths of the preparation in the Sham group and the PA-banding group were almost identical.
(TIF)

S3 Fig Photos of papillary muscles used in this study. All papillary muscles used in this study (stained with Masson’s trichrome staining).
(TIF)

S4 Fig Quantitative analysis of the interstitial fibrosis in long-axis section. We analyzed the interstitial fibrosis area examined by Masson’s trichrome staining in the long-axis sections of one-half of the papillary muscles from rats with Sham and PA-banding, as shown in Fig 1C (n = 4 in Sham, n = 4 in F-, and n = 4 in F+ 2 weeks after PA-banding; n = 12 in Sham, n = 6 in F-, and n = 3 in F+ 4 weeks after PA-banding; n = 12 in Sham, n = 13 in F-, and n = 3 in F+ 6 weeks after PA-banding).
(TIF)

S5 Fig Expression levels of Na_v_1.5 (SCN5A) in Sham, F- and F+. Expression of Na_v_1.5 mRNA was measured by QRT-PCR. Values are means ± SE; n = 21 in Sham, n = 19 in F-, and n = 6 in F+. Primers are designed as follows. SCN5A forward: GCTTCGCTTGAGGTCAGTGCTA. SCN5A reverse: TGCCACATCTCAGAAGCAAGCTA.
(TIF)
