# Supplementary figures and images for: Impairment of Excitation-Contraction Coupling in Right Ventricular Hypertrophied Muscle with Fibrosis Induced by Pulmonary Artery Banding
Source: PLoS One. 2017 Jan 9;12(1):e0169564. doi: 10.1371/journal.pone.0169564 (PMC5222608; doi:10.1371/journal.pone.0169564)

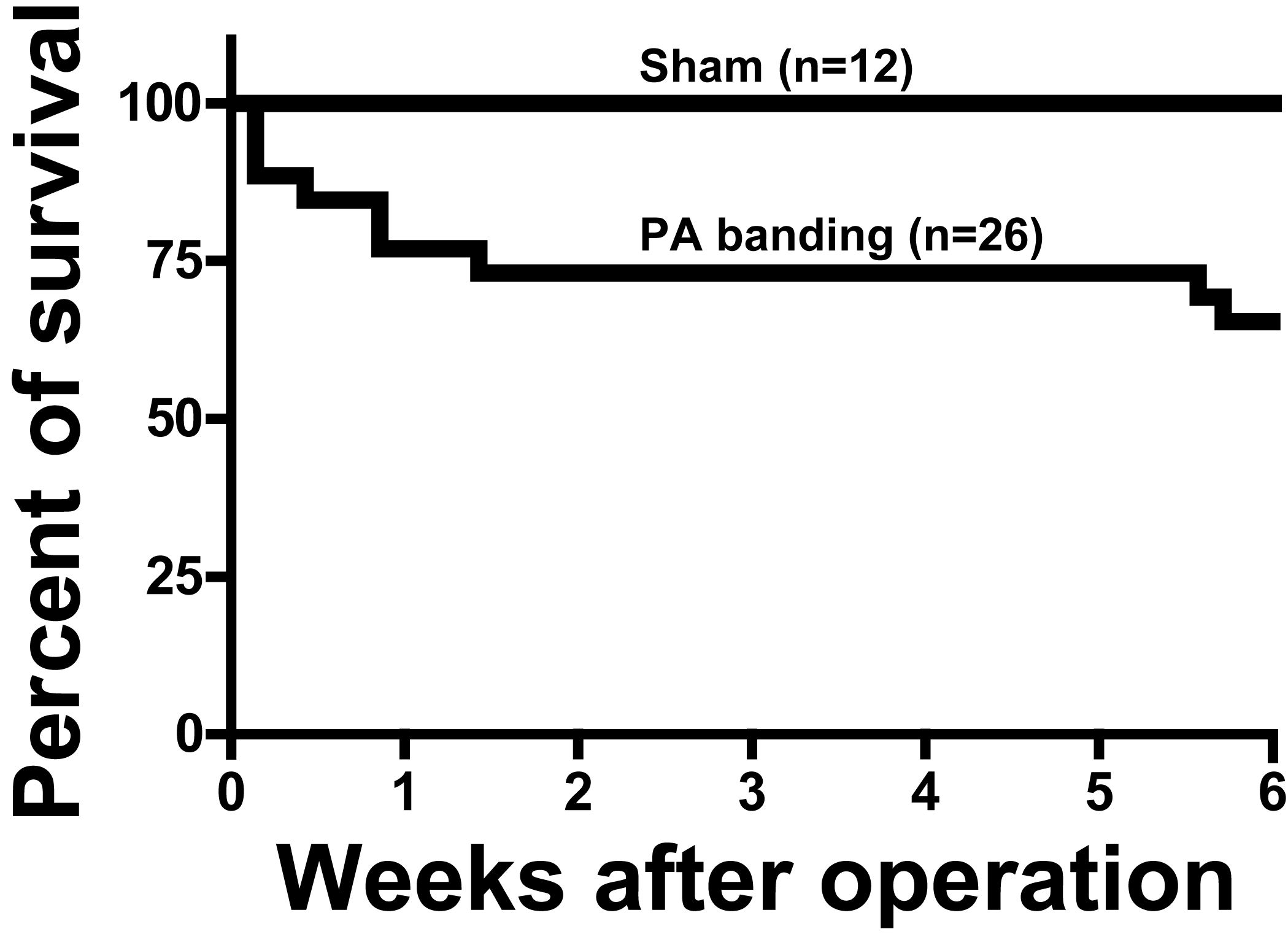

Supplement: S1 Fig — The Kaplan-Meier curve of PA-banding and Sham-operated rats is shown. The survival rate of rats with PA-banding was significantly lower than that of Sham-operated rats. (TIF) [file pone.0169564.s001.tif]

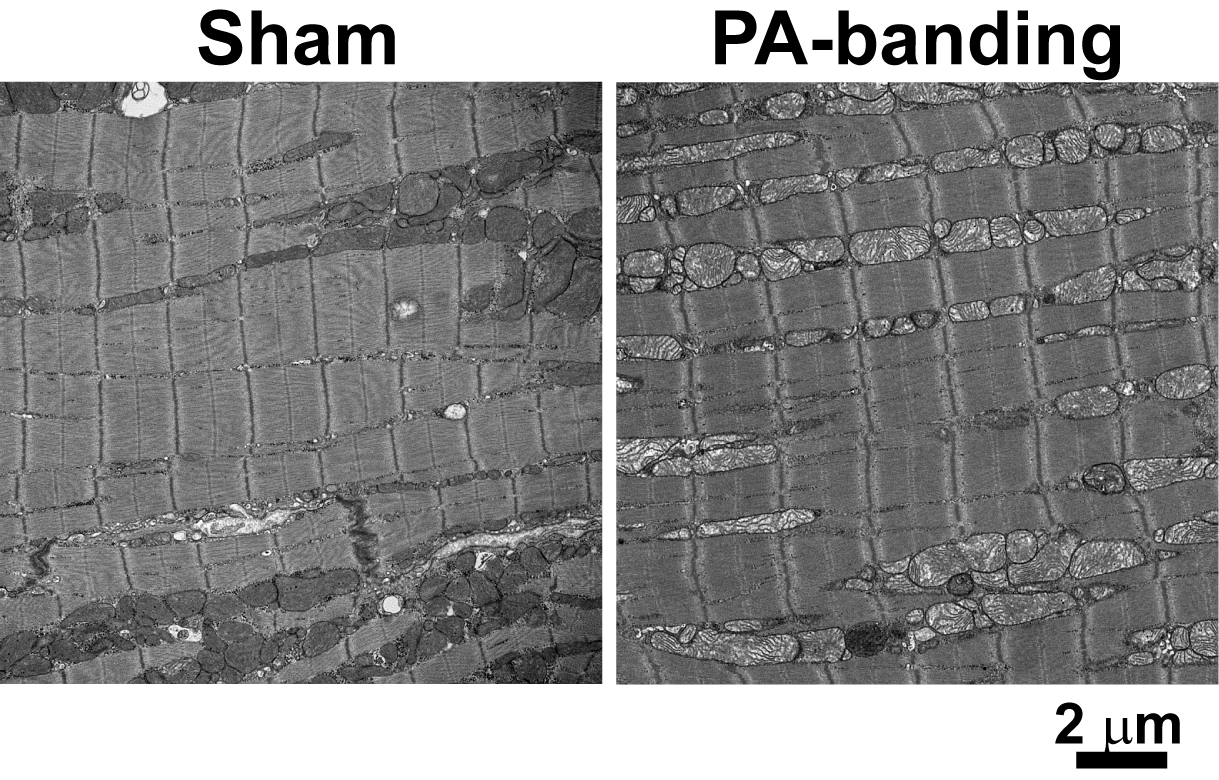

Supplement: S2 Fig — Representative electron microscope images are shown. At Lmax, the sarcomere lengths of the preparation in the Sham group and the PA-banding group were almost identical. (TIF) [file pone.0169564.s002.tif]

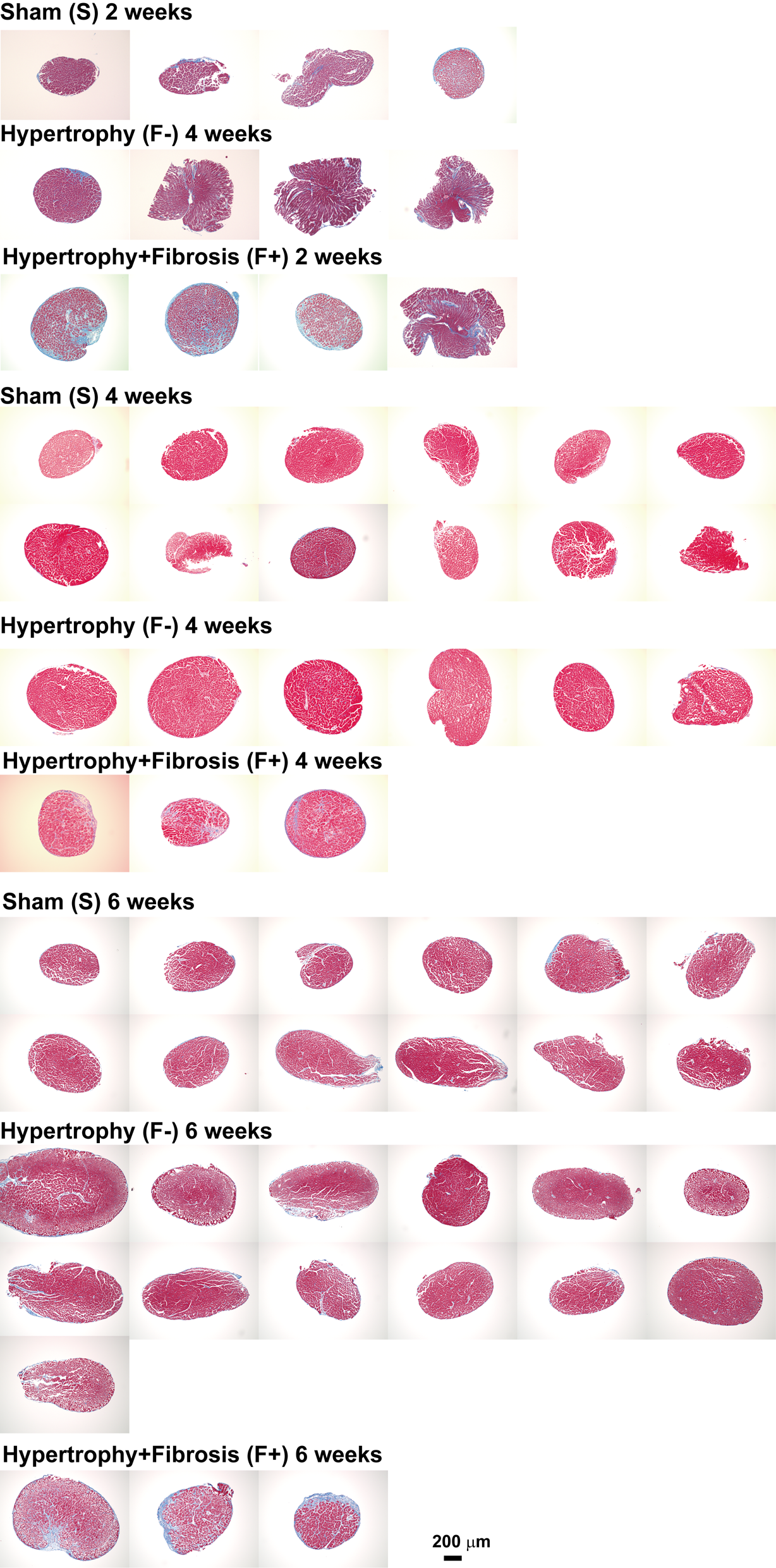

Supplement: S3 Fig — All papillary muscles used in this study (stained with Masson’s trichrome staining). (TIF) [file pone.0169564.s003.tif]

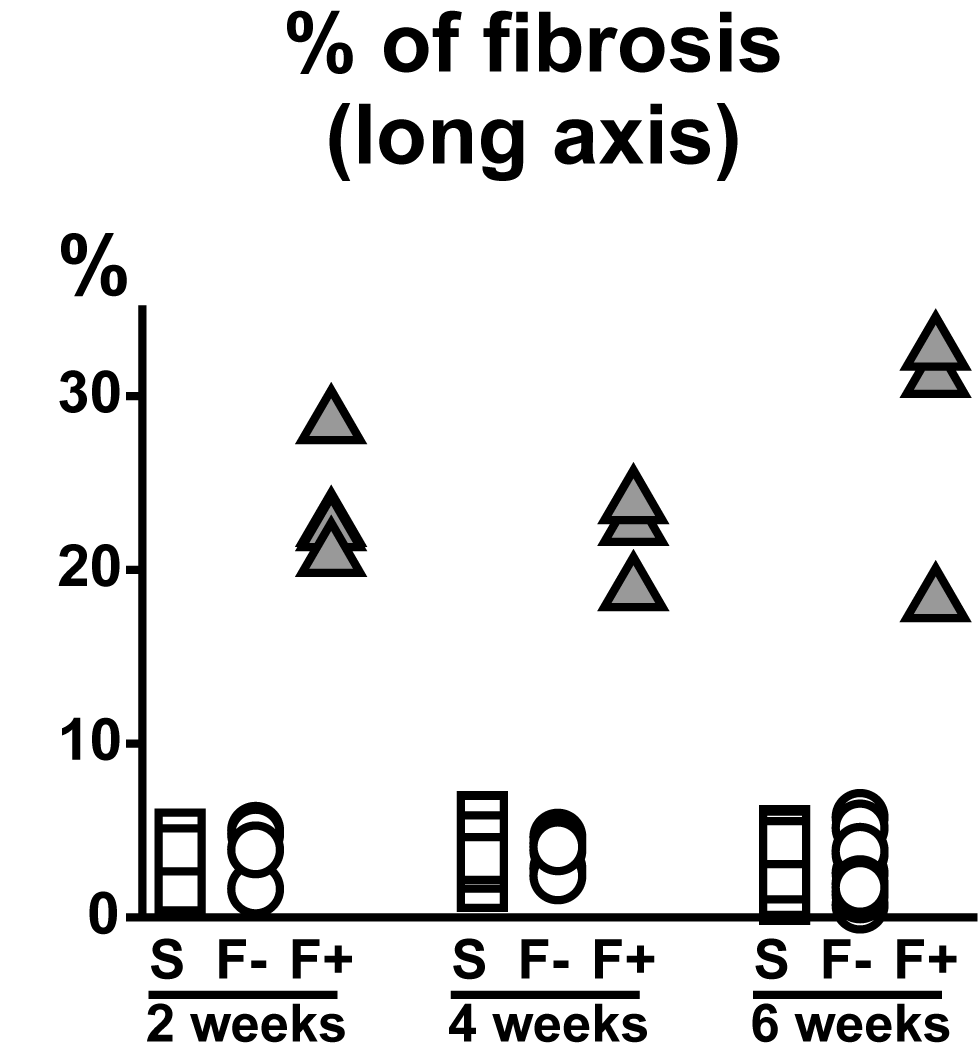

Supplement: S4 Fig — We analyzed the interstitial fibrosis area examined by Masson’s trichrome staining in the long-axis sections of one-half of the papillary muscles from rats with Sham and PA-banding, as shown in Fig 1C (n = 4 in Sham, n = 4 in F-, and n = 4 in F+ 2 weeks after PA-banding; n = 12 in Sham, n = 6 in F-, and n = 3 in F+ 4 weeks after PA-banding; n = 12 in Sham, n = 13 in F-, and n = 3 in F+ 6 weeks after PA-banding). (TIF) [file pone.0169564.s004.tif]

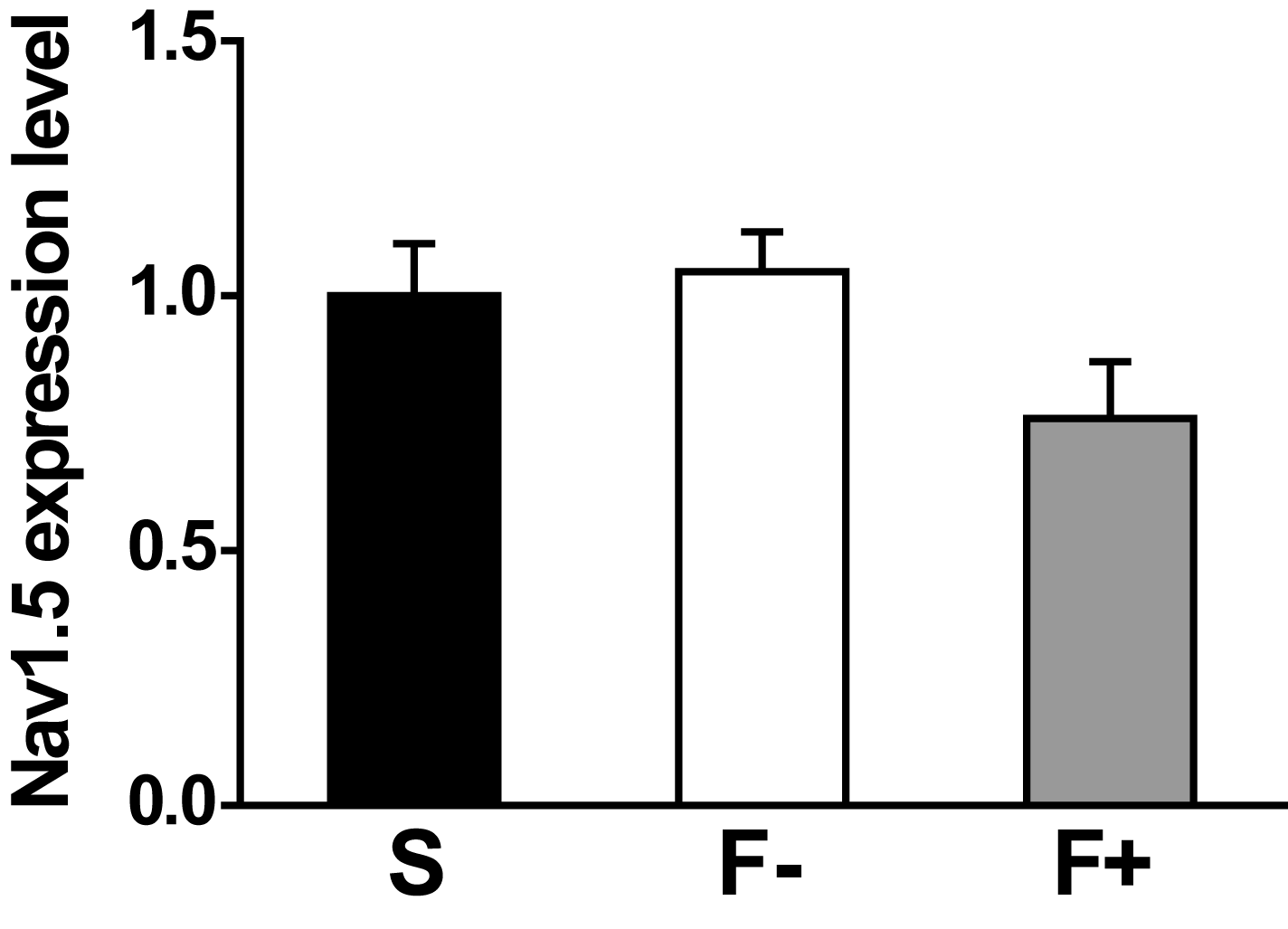

Supplement: S5 Fig — Expression of Nav1.5 mRNA was measured by QRT-PCR. Values are means ± SE; n = 21 in Sham, n = 19 in F-, and n = 6 in F+. Primers are designed as follows. SCN5A forward: GCTTCGCTTGAGGTCAGTGCTA. SCN5A reverse: TGCCACATCTCAGAAGCAAGCTA. (TIF) [file pone.0169564.s005.tif]
